# Statins in the Prevention and Treatment of Heart Failure: a Review of the Evidence

**DOI:** 10.1007/s11883-019-0800-z

**Published:** 2019-07-27

**Authors:** Matthew M. Y. Lee, Naveed Sattar, John J. V. McMurray, Chris J. Packard

**Affiliations:** Institute of Cardiovascular and Medical Sciences, University of Glasgow, Glasgow, Scotland

**Keywords:** Statin, Heart failure, Prevention, Coronary disease

## Abstract

**Purpose of Review:**

We summarize the best evidence for statins in the prevention and treatment of heart failure.

**Recent Findings:**

In patients with cardiovascular risk factors or established atherosclerotic cardiovascular disease (but without heart failure), statins reduce the risk of incident heart failure—mainly by preventing myocardial infarction although an additional benefit from reducing myocardial ischemia cannot be excluded. However, in patients with established heart failure, statins do not reduce the risk of cardiovascular death, which is mainly caused by pump failure and ventricular arrhythmias. Retrospective analyses, however, suggest that statins may reduce the rate of heart failure hospitalization and atherosclerotic events (which are proportionately much less common in these patients than heart failure hospitalization or death).

**Summary:**

Statin therapy should probably be continued in patients with coronary artery disease developing heart failure, although the weak evidence and small benefit may not justify the use of this treatment in very elderly patients with a short life expectancy and in which polypharmacy is a problem.

## Introduction

Heart failure (HF) is a clinical syndrome characterized by a constellation of symptoms that may be accompanied by signs caused by a structural and/or functional cardiac abnormality, resulting in a reduced cardiac output and/or elevated intracardiac pressures at rest or during stress [1]. Two principal HF phenotypes are recognized—HF with preserved ejection fraction (HFpEF) and HF with reduced ejection fraction (HFrEF) [1]. The predominant etiology of HFpEF is hypertension and patients with this phenotype are older, more often obese, and more likely to be women than patients with HFrEF. Compared with HFpEF, patients with HFrEF generally are younger, more often men, and more likely to have coronary artery disease [2, 3].

HF can be prevented by treatment of risk factors such as hypertension, obesity, and diabetes. Other treatments are also effective in slowing or preventing the development of HF after cardiac damage has occurred, e.g., the use of angiotensin-converting enzyme inhibitors after myocardial infarction (MI).

## Cholesterol Lowering in the Context of Heart Failure

There is a recognized “cholesterol paradox” in this cardiac condition. That is, although raised plasma cholesterol is a causal risk factor for coronary artery disease (CAD), low serum total cholesterol is associated with poor prognosis in patients with established HF (in contrast to patients without HF) [4–9]. The question is, of course, to what extent this counter-intuitive relationship represents “cause and effect.” Notably, cholesterol has an inverse correlation with disease severity markers in other diseases (e.g., chronic kidney disease, severe rheumatoid arthritis) which have a strong inflammatory component [10, 11]. Thus, possible reasons for the inverse association of cholesterol with HF are the finding that this lipid is known to be a marker of nutritional status (perhaps in part linked to greater systemic inflammation) in mild to moderate HF [12]. It is also possible that hepatic congestion (due to HF) could impair hepatic biosynthesis of cholesterol and so lower circulating levels since the liver is the main organ involved in lipoprotein production [5]. Another possible explanation is that HF causes intestinal congestion and therefore impairs cholesterol absorption, although this remains a hypothesis, with limited evidence [13]. In EVEREST, in 3957 patients hospitalized for worsening HF with left ventricular ejection fraction (LVEF) ≤ 40%, both total plasma cholesterol and triglyceride exhibited a significant inverse relationship to outcome [14], whilst follow-up of a cohort of 305 HF patients over 20 years showed that low low-density lipoprotein cholesterol (LDL-C) levels may predict a less favorable outcome, especially in patients < 70 years and those on statins [15]. Further, a lower high-density lipoprotein cholesterol is associated with worse prognosis in HF [16], as is low serum apolipoprotein A-I [17]. A German cohort study in 422 idiopathic dilated cardiomyopathy patients found that low cholesterol levels were dependent on the severity of cardiac disease [18], and a study of 288 very elderly patients hospitalized for medical conditions found that low LDL-C inversely correlated with N-terminal pro-B-type natriuretic peptide (NT-proBNP) [19]. The current working hypothesis is that lower plasma cholesterol is indeed secondary to the severity of patient’s condition rather than an independent risk factor for poor outcome [20, 21]. As noted above, this pattern is in line with other chronic conditions and thus observational data from people with such diseases must be interpreted with care.

## Statins in the Prevention of Cardiovascular Disease and Heart Failure

It is well established that HMG-CoA reductase inhibitors (statins) reduce CAD events in patients with and without diagnosed cardiovascular (CV) disease. Clinical trial outcome data have been subject exhaustively to meta-analysis that has reinforced the findings of individual studies and “filled in” any gaps in the determination of efficacy in sub-groups in the population [22, 23]. The main findings are the consistent demonstration that each 1.0 mmol/L reduction in LDL-C decreases the risk of major vascular events by 22% (Cholesterol Treatment Trialists’ (CTT) Collaboration which used individual level data on 170,000 in 26 trials) [24]. Ongoing concerns about statin therapy, particularly its safety, were addressed comprehensively in a meta-analysis in 2017 [25]. Further, there have been recent expert reports from The European Atherosclerosis Society (EAS) [26] and American Heart Association [27] on the issue of muscle-related side effects addressing their incidence rate and impact on adherence. To bolster the evidence base for statin therapy, extended follow-up data documenting safety and efficacy has become available for a number of trials including WOSCOPS [28, 29], ASCOT-LLA [30], and PROSPER which showed that even in the elderly, coronary protection was maintained in the long-term (mean follow-up 8.6 years) [31]. There is, however, a relative dearth of data from the major lipid-lowering trials on the effects of statin therapy on prevalent HF since most trials excluded patients with this syndrome [4, 32, 33]. For example, 4S [34] and LIPID [35] excluded patients with HF altogether, whilst CARE [36] and HPS [37] included only patients with mild to moderately severe symptoms and excluded those with severely symptomatic HF. HPS showed smaller reductions in major vascular events among those with greater baseline natriuretic peptide levels [38].

**Fig. 1:**
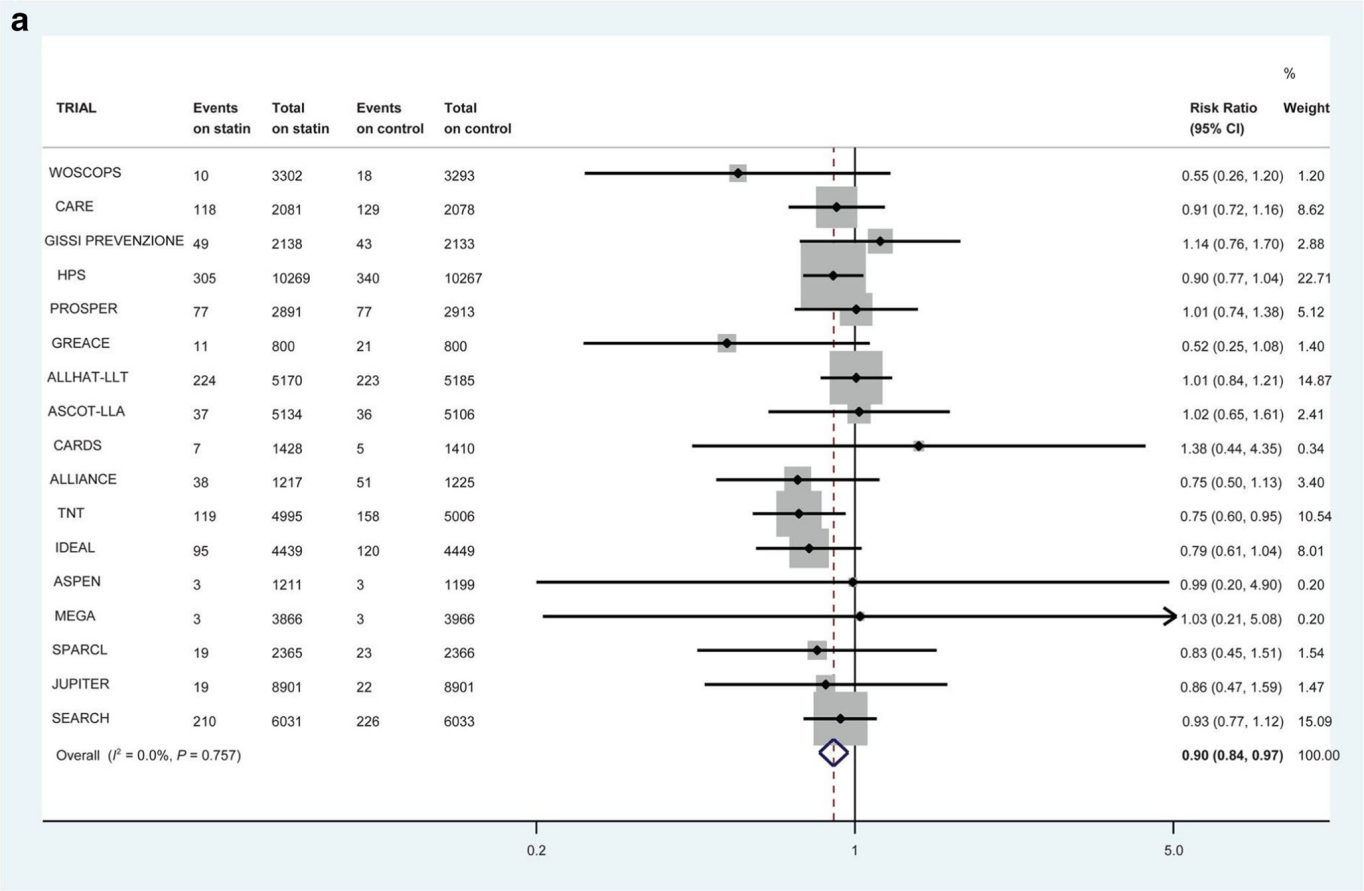

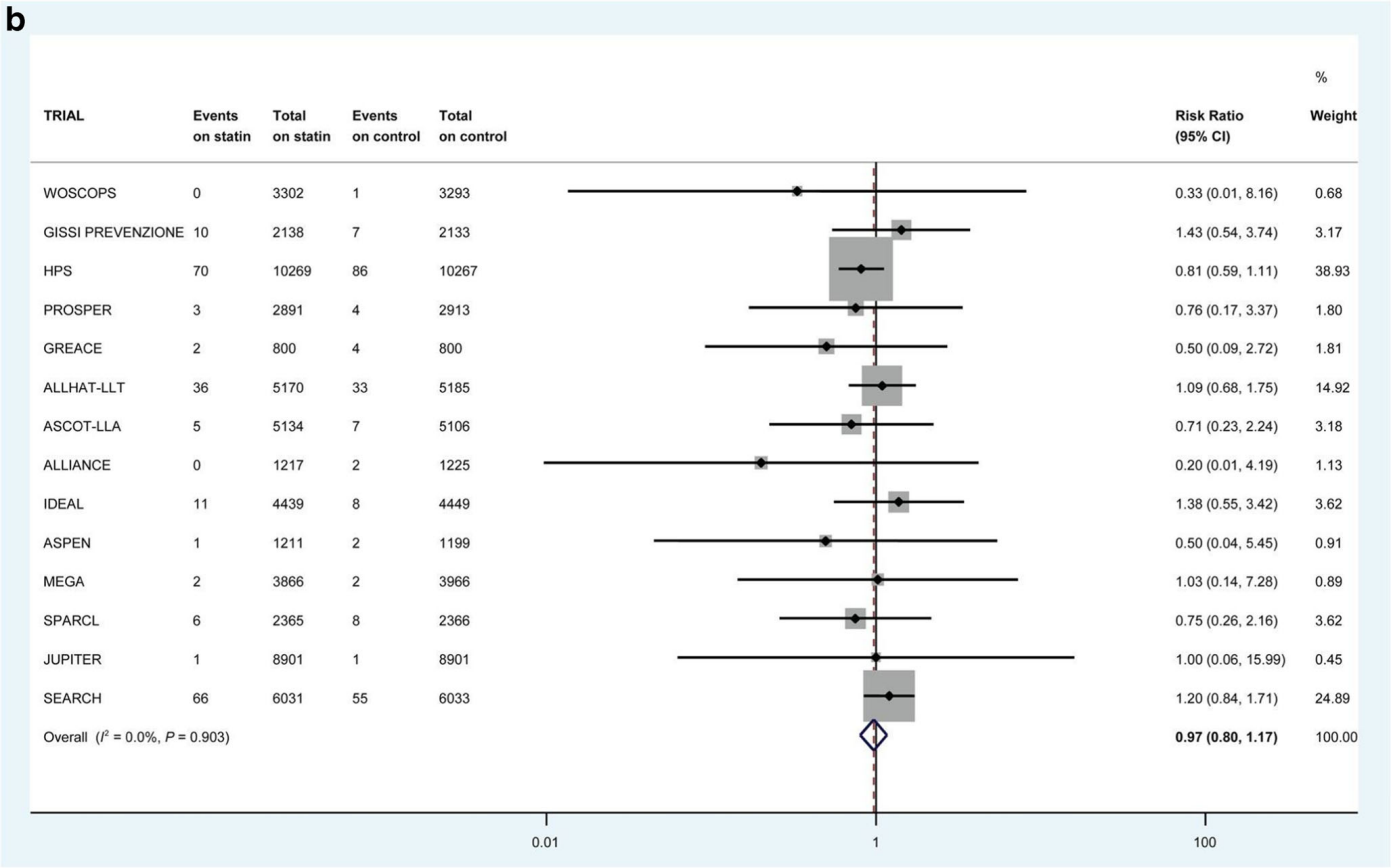

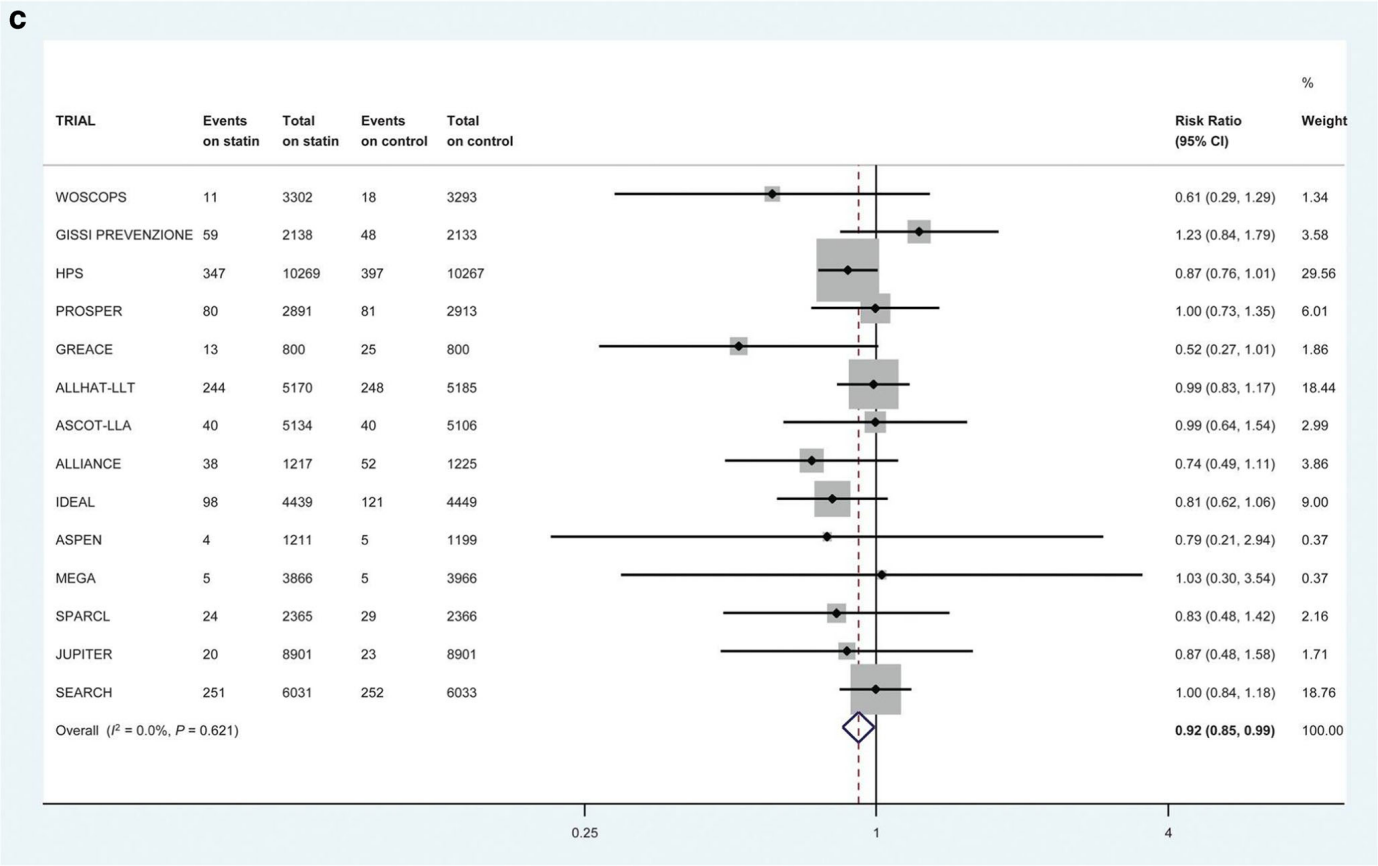
The effect of statin therapy on the risk of first non-fatal heart failure hospitalization in 17 trials (**a**), heart failure death in 14 trials (**b**), and first composite heart failure outcome in 14 trials (**c**). All heart failure events within 30 days of myocardial infarction were excluded. Reproduced (as permitted by terms of the CreativeCommonsAttribution License) (http://creativecommons.org/licenses/by/4.0/) from Preiss D, Campbell RT, Murray HM, Ford I, Packard CJ, Sattar N, et al. “The effect of statin therapy on heart failure events: a collaborative meta-analysis of unpublished data from major randomized trials.” *Eur Heart J*. 2015;36:1536–46

Some of these trials do allow us to look at incident heart failure. Collectively, these trials provide “reasonable evidence” that statin use can prevent or delay the onset of HF [39–41]. This therapeutic link is based on the supposition that statins prevent MI and consequent cardiac damage and so reduce the risk of developing reduced ejection fraction HF. A meta-analysis of 6 randomized controlled trials (RCTs) of 110,271 patient-years in patients with recent acute coronary syndrome (ACS) showed that intensive statin therapy reduced hospitalization rates for HF [40]. Further, in a larger collaborative meta-analysis of up to 17 major primary- and secondary-prevention randomized trials with 132,538 participants conducted over 4.3 years, statins modestly reduced the risks of non-fatal hospitalization for HF (Fig. 1a) and the composite of non-fatal hospitalization or death from HF (Fig. 1c) but not HF death (Fig. 1b) [42••]. There was no demonstrable difference in risk reduction between those who suffered an incident MI or not [42••]. Interestingly, therefore, this benefit did not seem to be due to prevention of MI preceding HF.

**Fig. 2:**
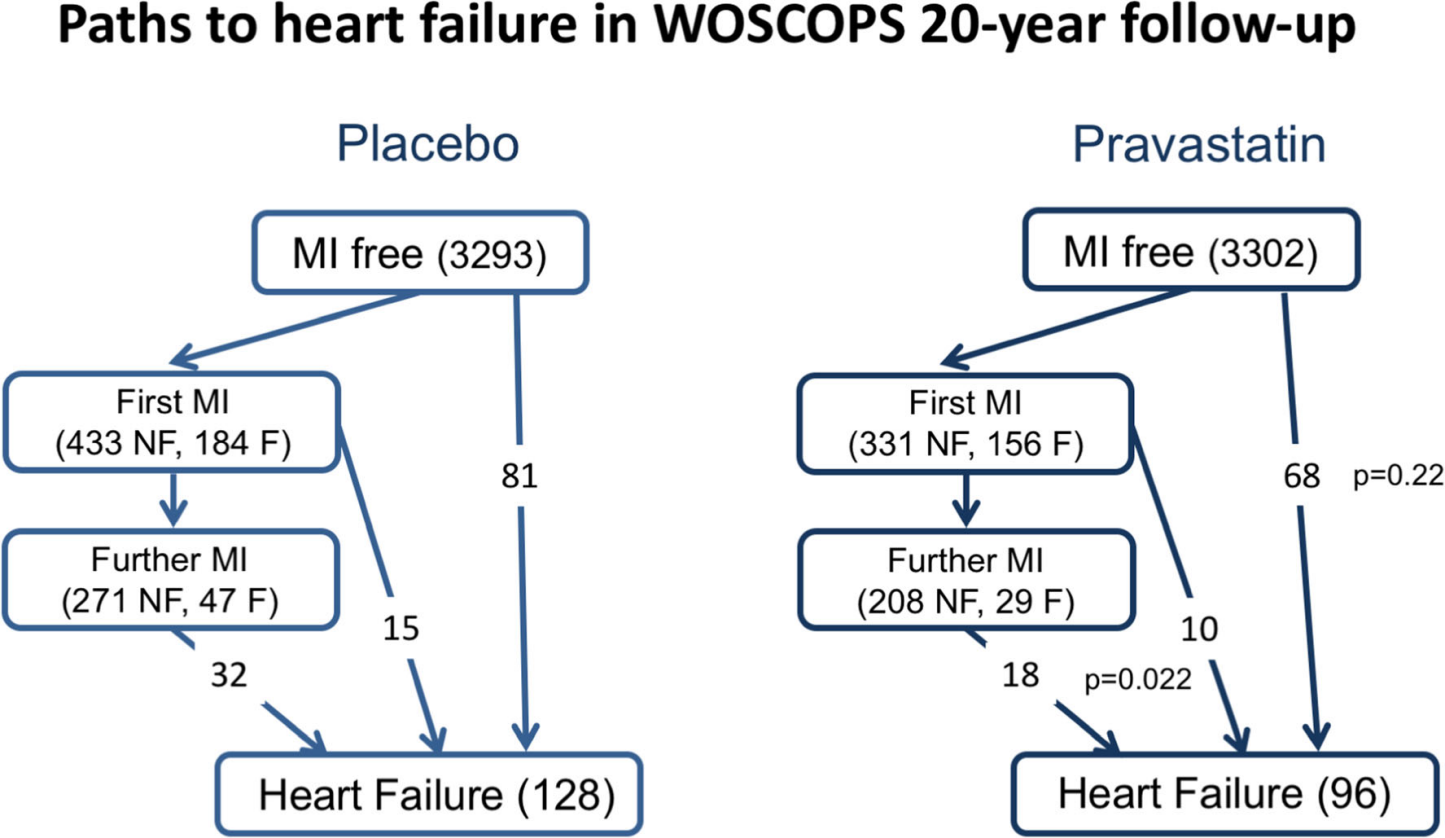
In WOSCOPS, after 20 years of follow-up, of the total of 224 subjects hospitalized for, or dying from, HF, 75 (33%) had incident MI preceding HF, whilst 149 (67%) did not. HF events subsequent to MI were reduced by pravastatin treatment, compared with placebo (28 vs 47) (*p* = 0.022). Incident HF not preceded by MI was also less common in pravastatin-treated patients (68 vs 81), although the difference was not significant (*p* = 0.22). Abbreviations: F, fatal; HF, heart failure; MI, myocardial infarction; NF, non-fatal; WOSCOPS, West of Scotland Coronary Prevention Study. Footnote: In subjects who experienced HF, the proportion with “antecedent MI” vs “no antecedent MI” does NOT differ between placebo and pravastatin groups (*p* = 0.24)

Retrospective examination of individual trials also provides supportive evidence for a beneficial effect of statins on HF prevention. In 4S, simvastatin reduced incident HF by 19% [41], whilst in the post-ACS studies (PROVE IT-TIMI 22), intensive statin therapy reduced the rate of hospitalization for HF (especially if subjects had elevated baseline B-type natriuretic peptide (BNP)) [39], and in the TNT trial in patients with stable CAD, hospitalization for HF was lower in the intensive statin treatment arm [43]. Even in the primary prevention setting, if viewed over a sufficiently long time frame, statin therapy reduced the incidence of HF endpoints. In WOSCOPS, an initial 5 years of statin therapy led to a 35% reduction in the long-term (20-year) risk of hospitalization for HF [44••]. Of the total of 224 subjects hospitalized for, or dying from, HF, 75 (33%) had incident MI preceding HF, whilst 149 (67%) did not (Fig. 2). HF events subsequent to MI were reduced by pravastatin treatment, compared with placebo (28 vs 47) (*p* = 0.022). Incident HF not preceded by MI was also less common in pravastatin-treated patients (68 vs 81), although the difference was not significant (*p* = 0.22). This is consistent with the finding in the meta-analysis above that statins reduce HF risk through mechanisms additional to prevention of acute MI. Serial measurement of biomarkers such as NT-proBNP might have provided more support for this hypothesis but, unfortunately, NT-proBNP was measured only at 1 year after randomization in WOSCOPS [45].

## Statins in the Treatment of Established Heart Failure

Cholesterol-lowering therapy with statins is not indicated in patients with moderate to severely symptomatic HF (New York Heart Association (NYHA) class III-IV) according to the 2011 European Society of Cardiology (ESC)/EAS dyslipidemia guideline [46] and the updated 2016 ESC HF Guidelines do not recommend initiation of statins in most patients with HF, but continuation may be considered for those already on statins for prevention of CAD [1]. These recommendations are based largely on the findings of two major outcome studies CORONA [47] and GISSI-HF [48] that were large-scale placebo-controlled trials of statin treatment in subjects with NYHA class II–IV HF. CORONA recruited 5011 ischemic systolic HF patients aged ≥ 60 years, whilst GISSI-HF included 4574 HF patients of any etiology aged ≥ 18 years. The active treatment arm used 10 mg/day rosuvastatin in both trials, and median follow-up was 46.8 months in GISSI-HF and 32.8 months in CORONA. In GISSI-HF, the co-primary endpoints were time to death, and time to death or CV hospitalization. In CORONA, the primary endpoint was the composite of CV death, non-fatal MI, or non-fatal stroke. No significant decrease in the primary composite mortality/morbidity endpoint was seen in actively treated subjects in either trial [47, 48]. However, CORONA did show that rosuvastatin therapy led to fewer CV hospitalizations [47], and in retrospective sub-group analyses, rosuvastatin appeared to provide more CV benefit in those with higher C-reactive protein [49] and lower baseline NT-proBNP [50], and YKL-40 (chitinase-3-like protein 1) levels [51]. When repeat events were included in the CORONA endpoint analysis, rosuvastatin reduced hospitalization for HF by 15–20%, but this was a post hoc analysis [52].

Not satisfied with the above trial evidence, a number of investigators have attempted to examine if statin therapy has any role in patients with established HF by examining randomized trials in a number of meta-analyses. For example, Zhang et al. reported no significant reduction from statin therapy for all-cause death, CV death, or rehospitalization for HF, but a non-significant trend to lower non-fatal MI [53]. Notably, however, CAD events are relatively uncommon in HF in general, even in ischemic HF, and are a minor contributor to the overall morbidity and mortality in patients with this condition [54]. The same group also investigated whether statin therapy had an effect on measures of left ventricular size and function, this time collating 11 RCTs totaling 590 patients [55]. Here they reported that statin use increased LVEF by 3% but notably, some of the trials were of modest quality, and there was considerable heterogeneity in the findings, so definitive conclusions cannot be made. Thus, whilst the best evidence suggests statins do not improve outcomes in patients with HF, clinicians generally continue HF patients on statins as most are not comfortable stopping them, unless statin-related complications arise.

## Potential Mechanisms by Which Statins Prevent HF Development

**Fig. 3:**
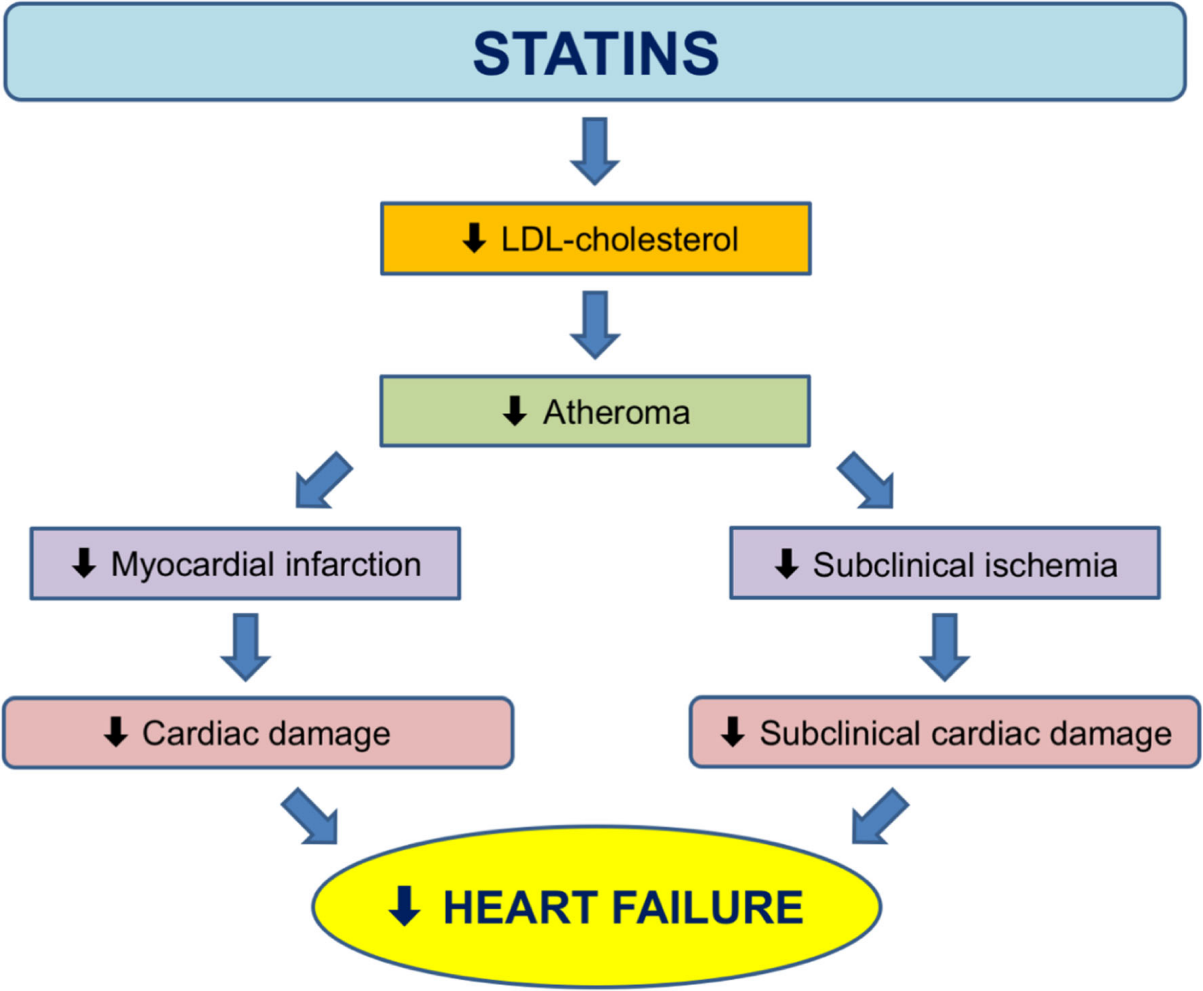
Proposed mechanistic pathways on how statins reduce heart failure: (1) by reducing myocardial infarction; (2) by reducing subclinical ischemia (e.g., statins lower troponin levels in WOSCOPS). Abbreviations: LDL, low-density lipoprotein

Statins are believed to lower CAD by stabilizing atheromatous plaques, reduction of atheroma volume, and prevention of formation of new atherosclerotic lesions resulting in fewer MIs and less associated cardiac tissue damage. This may be the main way statins lower incident HF. We looked further at this in the long-term follow-up of WOSCOPS (Fig. 2) where, as noted before, it appeared that statin use decreased the incidence of HF. In a companion report, we found that pravastatin reduced high-sensitivity troponin I concentration by 13% [56•]—and troponins also predict incident HF. Thus, it is theoretically possible that statins lessen “subclinical” ischemia and cardiac damage (Fig. 3). Whilst others have proposed a number of pleiotropic actions of statins (e.g., anti-inflammatory), there is no clear evidence for this contributing to clinical benefit [57].

## Non-Statin Lipid-Lowering Therapies in the Prevention of Heart Failure

PCSK9 inhibitors have not been found to affect HF hospitalisation (OR 0.98; 95% CI 0.86–1.13; *P* = 0.79) in a meta-analysis of 23 RCTs of 42,151 participants [58]. One ezetimibe randomized trial (HIJ-PROPER) of patients with acute coronary syndrome and dyslipidemia showed that HF hospitalisation was reduced in the intensive (pitavastatin + ezetimibe) vs. standard (pitavastatin monotherapy) lipid-lowering group, although the number of events was small (*n* = 59) and the 95% confidence interval around the estimate of treatment effect wide (HR 0.47; 95% CI 0.27–0.81; *P* = 0.006) [59]. The ACCORD and ACCORDION trial of fenofibrate showed no significant effect on congestive HF [60]. There are no robust data of the effect of niacin on HF.

## Conclusions and Clinical Implications

There is robust evidence that in addition to lowering coronary heart disease (CHD), statins also lower risk for incident HF both in the medium and long term, although the extent of risk reduction is modest and far lower than the comparable benefits on CHD outcomes. By contrast, in patients with established HF, best evidence from two landmark trials does not support improved clinical outcomes with statins. Although statins may also lessen the chances of subsequent CAD events in HF patients (for which there is limited evidence in a meta-analysis of RCTs), few patients with HF actually die from acute CAD events and existing evidence is far from conclusive. Nevertheless, most clinicians generally do not stop statins in patients already prescribed these drugs and who subsequently develop HF. However, more judicious use of statins in the elderly HF patient with polypharmacy and a shorter life expectancy appears to be sensible.
